# The Effect of Web-Based Culinary Medicine to Enhance Protein Intake on Muscle Quality in Older Adults: Randomized Controlled Trial

**DOI:** 10.2196/49322

**Published:** 2024-02-13

**Authors:** Emily Salas-Groves, Michelle Alcorn, Allison Childress, Shannon Galyean

**Affiliations:** 1 Nutritional Sciences Texas Tech University Lubbock, TX United States; 2 Hospitality and Retail Management Texas Tech University Lubbock, TX United States

**Keywords:** older adults, culinary medicine, protein, muscle mass, muscle strength, physical activity, nutrition intervention, online

## Abstract

**Background:**

The most common age-related musculoskeletal disorder is sarcopenia. Sarcopenia is the progressive and generalized loss of muscle mass, strength, and function. The causes of sarcopenia can include insufficient nutritional status, which may be due to protein-energy malnutrition, anorexia, limited food access and eating ability, or malabsorption. In the United States, 15.51% of older adults have been diagnosed with sarcopenia. Culinary medicine (CM) is a novel evidence-based medical field that combines the science of medicine with food and cooking to prevent and treat potential chronic diseases. CM helps individuals learn and practice culinary skills while tasting new recipes. Therefore, this program could successfully reduce barriers to protein intake, enabling older adults to enhance their diet and muscle quality.

**Objective:**

This study aimed to examine how a web-based CM intervention, emphasizing convenient ways to increase lean red meat intake, could improve protein intake with the promotion of physical activity to see how this intervention could affect older adults’ muscle strength and mass.

**Methods:**

A 16-week, single-center, parallel-group, randomized controlled trial was conducted to compare a web-based CM intervention group (CMG) with a control group (CG) while monitoring each group’s muscle strength, muscle mass, and physical activity for muscle quality. The CMG received weekly web-based cooking demonstrations and biweekly nutrition education videos about enhancing protein intake, whereas the CG just received the recipe handout. Anthropometrics, muscle mass, muscle strength, dietary habits, physical activity, and cooking effectiveness were established at baseline and measured after the intervention. The final number of participants for the data analysis was 24 in the CMG and 23 in the CG.

**Results:**

No between-group difference in muscle mass (*P*=.88) and strength (dominant *P*=.92 and nondominant *P*=.72) change from the prestudy visit was detected. No statistically significant difference in protein intake was seen between the groups (*P*=.50). A nonsignificant time-by-intervention interaction was observed for daily protein intake (*P*=.08). However, a statistically significant time effect was observed (*P*≤.001). Post hoc testing showed that daily protein intake was significantly higher at weeks 1 to 16 versus week 0 (*P*<.05). At week 16, the intake was 16.9 (95% CI 5.77-27.97) g higher than that at the prestudy visit.

**Conclusions:**

This study did not affect protein intake and muscle quality. Insufficient consistent protein intake, low physical activity, intervention adherence, and questionnaire accuracy could explain the results. These studies could include an interdisciplinary staff, different recruitment strategies, and different muscle mass measurements. Future research is needed to determine if this intervention is sustainable in the long term and should incorporate a follow-up to determine program efficacy on several long-term behavioral and health outcomes, including if the participants can sustain their heightened protein intake and how their cooking skills have changed.

**Trial Registration:**

ClinicalTrials.gov NCT05593978; https://clinicaltrials.gov/ct2/show/NCT05593978

## Introduction

The guidance of the National Institute on Aging classifies older adults as those aged 65 years and older [1]. As adults age, several age-related diseases can occur, the most common being cardiovascular disease, cancer, Alzheimer disease, Parkinson disease, osteoporosis, and sarcopenia [2]. A Global Burden of Disease study in 2017 [3] revealed that 31.4% of all diseases were age related. These age-related diseases, combined with the body and life changes that occur with aging, could contribute to compromised nutritional status. These body and life changes can be physiological, psychosocial, and economic [4]. All these factors play a significant role in nutrition and food choices, which are barriers to appetite and diet quality. Therefore, current research strategies aim to acquire healthy aging and prevent age-related diseases.

Aging can lead to age-related musculoskeletal disorders [5] caused by an imbalance between muscle protein’s anabolic and catabolic pathways, leading to overall skeletal muscle mass (SMM) loss [6]. The most common age-related musculoskeletal disorder is sarcopenia. Sarcopenia is the progressive and generalized loss of muscle mass, strength, and function [2,7,8]. Muscles affected include skeletal [9], smooth [10], and cardiac [11]. Consequently, sarcopenia increases fall and fracture risk [12], impairs daily living activities performance [13], increases cognitive impairment [14], decreases the quality of life [15], and leads to death [16].

In research, the general sarcopenia prevalence ranges from 0.2% to 86.5%, with prevalence in women ranging from 0.3% to 91.2% and prevalence in men ranging from 0.4% to 87.7% [17]. In the United States, 15.51% of older adults have been diagnosed with sarcopenia, demonstrating its magnitude of being a public health burden [18]. Therefore, early identification and intervention are the key factors for achieving improved sarcopenia outcomes. According to the European Working Group on Sarcopenia in Older People (EWGSOP), a sarcopenia diagnosis requires the measurements of muscle mass, strength, and function [6].

Although many factors lead to sarcopenia, the 2 crucial factors that can be controlled in older adults are inadequate nutritional intake and physical inactivity [19,20]. Older adults tend to have anabolic resistance, defined as “a blunted stimulation of muscle protein synthesis (MPS) to common anabolic stimuli in SMM” [21]. Therefore, increasing protein-dense food ingestion and habitual physical activity are frontline strategies to support muscle mass, performance, and health [21]. The Society for Sarcopenia, Cachexia, and Wasting Disease provided protein recommendations for treating and preventing sarcopenia at a minimum of 1.0 to 1.5 g/kg body weight per day with exercise [22]. The protein quality is also critical in age-related SMM anabolism. Research on how protein-rich whole foods (eg, lean red meat) can enhance MPS over supplementation in older adults is rising [23]. Recent data suggest that a moderate 113 g (30 g of protein) serving of animal protein (eg, lean beef) can increase MPS by approximately 50% [24]. Therefore, the per-meal anabolic threshold recommendation is 25 to 30 g of protein [23-25]. Unfortunately, older adults’ protein needs are usually not met. Independent older adults answered the 2005-2014 National Health and Nutrition Examination Survey (NHANES) [26], revealing that up to 46% are not meeting the protein intake recommendation.

Physical activity directly impacts muscle quality and quantity [27]. Inactivity in older adults can promote sarcopenia development [28,29], whereas physical activity increases muscle strength [30,31] and mass [32,33]. Therefore, physical activity is vital to lower sarcopenia prevalence [34-36]. Specifically, resistance training and balance exercises are considered the best for sarcopenia prevention [27,37-41]. Steps through activity trackers can help determine one’s physical activity [42]. Accomplishing 10,000 daily steps is suggested to positively influence body composition (eg, weight and body fat) and improve health parameters (eg, quality of life) [43]. Therefore, nutrition and physical activity have been seen to be essential in countering sarcopenia [44].

More interventions focusing on nutrition and lifestyle changes are essential in decreasing chronic disease and health care costs [45]. Educating and empowering individuals to change their lifestyles can be less costly than medications and invasive procedures [45]. Culinary medicine (CM) is a novel evidence-based medical field defined by combining the science of medicine with food and cooking [46]. CM differs from traditional lifestyle and nutrition interventions by attempting to empower the patient to care for herself or himself safely, effectively, and happily with food and beverages as a primary care technique [47]. It helps people access and eat nutrient-dense meals to prevent and treat potential chronic diseases [46]. Individuals learn and practice culinary skills while tasting new recipes [45]. Also, they can incorporate their favorite foods into their eating plan while learning how to enhance diet quality through new foods (eg, different types of vegetables) and meal preparation tips (eg, defrosting techniques) [47,48]. If executed appropriately, CM can be taught to all populations regardless of culinary skill, educational level, or socioeconomic background [45]. A CM curriculum typically includes practical applications in supermarkets and home kitchens [49]. These practical applications include basic nutrition knowledge and instruction on how to apply that knowledge to diet therapies [49]. However, limited studies report whether a web-based CM curriculum could be as effective as in-person.

Multiple randomized controlled trials report that CM significantly improved individuals’ culinary knowledge, healthy dietary patterns, and self‐efficacy for healthier cooking [50-54]. Thus, highlighting CM’s potential as a nutrition intervention could lower the risk of diet‐related chronic disease among older adults. However, few studies in this area include older adult participants; none exclusively focused on an older adult population, and only 6% of CM programs were taught by a qualified health professional [55]. Additionally, CM interventions have been very heterogeneous, indicating a lack of variety in how the intervention is conducted compared with others [55]. Therefore, this study could advance our knowledge of CM and sarcopenia prevention in older adults. A web-based CM program might be an innovative strategy to improve protein intake in independent older adults at home. In addition, this program could successfully reduce barriers to protein intake, enabling older adults to enhance their diet and muscle quality. This factor could be vital because research surrounding CM within older adults is in its infancy. Therefore, our study aimed to examine how a web-based CM intervention, emphasizing convenient ways to increase lean red meat intake, could improve protein intake with the promotion of physical activity to see how this intervention could affect older adults’ muscle strength and mass.

## Methods

### Study Design

A 16-week, single-center, parallel-group, randomized controlled trial compared a web-based CM intervention group (CMG) with a control group (CG) on their protein intake, cooking effectiveness, muscle strength, muscle mass, and physical activity. The study was conducted at Texas Tech University Nutrition and Metabolic Health Initiative (NMHI), Lubbock, Texas. Participants were permitted to remove themselves from the trial at any time.

### Ethical Considerations

A human study compliance review was submitted to the institutional review board at Texas Tech University, Lubbock, Texas. The study was expedited for review and received approval (IRB2021-693). Once participants were recruited and eligibility was determined, an initial appointment was set up at Texas Tech University NMHI. A research team member described the study in detail, and participants were asked to sign a consent form stating willingness to participate. The participants’ information collected for the study was deidentified, given a code number, and kept on the researchers’ computer at Texas Tech University NMHI. The research team offered the participants the vívofit 4 watch (Garmin) as compensation, which they used to complete the study.

### Recruitment, Screening, and Participants

Flyers, newsletters, and word of mouth were essential for recruitment. When participants agreed to enroll in the study, they filled out an initial screening questionnaire to help determine whether they met the eligibility criteria. The inclusion criteria involved individuals who are aged 65 years or older, able to cook for themselves, physically active (eg, no need for equipment for assistance), and able to use a computer and mobile device. The exclusion criteria included individuals aged <65 years; those with limited mobility (eg, need for equipment for assistance), cognitive dysfunction (eg, dementia), a heart pacemaker, or type 1 or type 2 diabetes with insulin use; current smokers; those with some form of amputation; those who unable to use a computer and mobile device or unable or unwilling to wear the vívofit 4 watch (Garmin) for the duration of the study; and those undergoing or had recently undergone a severe medical procedure or diagnosis.

Participants were recruited and enrolled from June 2022 to August 2022, with data collection completed in December 2022. If a participant dropped out of the study, a new participant would replace and be allotted to the same group as the participant they replaced. A total of 52 older adults, including both men and women, met the study’s eligibility criteria. Assessments were conducted at the prestudy, weekly, and poststudy time points.

### Intervention Design and Study Procedures

#### Prestudy Visit

Before their visit, participants were told to refrain from exercising for 48 hours, taking alcohol for 12 hours, and wearing clothes with any metals. Informed consent was obtained before starting the assessments. The assessments included completing 4 questionnaires: Community Healthy Activities Model Program for Seniors (CHAMPS), Dietary Screener Questionnaire (DSQ), protein questionnaire, and cooking effectiveness questionnaire. Afterward, grip strength, height, and weight were measured. Then, the participants were scanned by dual-energy x-ray absorptiometry (DXA). After completing their scan, they were given a vívofit 4 watch (Garmin). Lastly, the participants were randomized to either CMG or CG and provided their study’s subject code (eg, Beef Study 01), grip strength and DXA results, and exercise handouts. Both groups were advised to consume 25 to 30 g of protein during every meal, and all questions were answered. A follow-up email was sent providing a sample of a 2-week workout plan based on the exercise recommendation handouts and reminders of the study protocol.

#### Weekly Interventions

The CMG received weekly web-based cooking demonstrations with a recipe handout and biweekly nutrition education video on general nutrition information based on the *Nutrition Care Manual* content from the Academy of Nutrition and Dietetics [56], all provided by email at the beginning of each week. Meanwhile, the CG just received the recipe handout by email. Therefore, this intervention was developed to show how effective the hands-on and visual intervention provided to the CMG is compared with just general reading of a recipe with no further education provided to the CG. In addition, at the end of each week, both groups received their weekly protein and cooking effectiveness questionnaires.

A total of 20 recipes focusing on lean ground beef were provided for this study. Before starting the study, the research team tested each recipe and adjusted it as needed based on visual, flavor, and dish size. Then, the cooking demonstration was recorded once the recipe was approved for the study. For weeks 1 and 2, three recipes were sent to the participants. For the remainder of the study, 1 recipe was sent weekly. In addition, educational videos on a specific nutrition topic were sent every 2 weeks. These topics provided the participants with further nutrition education, which is essential regarding their diet outside of protein.

#### Poststudy Visit

After their 16th week, the participants had their final data collected. At the end of the visit, the primary researcher shared the pre- and poststudy DXA and grip strength results with the participant and answered any questions.

### Outcome Measurements

#### Questionnaires

The following outcomes were measured: weekly activity level through CHAMPS, the diet through the DSQ, protein intake through a protein questionnaire, cooking confidence and attitude using a pre- and poststudy cooking effectiveness questionnaire, and intervention compliance through weekly cooking effectiveness.

CHAMPS is a 41-item questionnaire [57] that assesses the weekly frequency and duration of various lifestyle physical activities that are appropriate for older adults. The DSQ was developed for the 2009-2010 NHANES [58]. It is a 30-item questionnaire that assesses the frequency of consumption of selected foods and drinks in the past month, such as intakes of fruits and vegetables, red and processed meat, dairy or calcium products, added sugars, and whole grains or fiber. The protein questionnaire is a modified version of the rapid self-administered dietary protein food frequency questionnaire, which contains 37 items evaluating the weekly intake of different types of meat, dairy products, eggs, and beans [59].

Lastly, the pre- and poststudy cooking effectiveness questionnaires measured participants’ cooking confidence, attitudes, and challenges or barriers. In addition, the weekly cooking effectiveness reported each group’s compliance toward their intervention. The prestudy cooking effectiveness questionnaire includes 14 items, the weekly cooking effectiveness questionnaire includes 5 items, and the poststudy cooking effectiveness questionnaire includes 33 items.

#### Anthropometrics

Height was measured using a Charder HM: 200P stadiometer (Charder Electronic Co Ltd) to the nearest half inch. Body weight was measured by a Brecknell MS-1000 wheelchair scale (Brecknell) to the nearest 0.5 lbs.

#### Muscle Quality

Lean body and fat mass were measured using a Norland XR-800 DXA (Swissray International, Inc) to the nearest gram. Muscle strength was measured by a Camry Digital Hand Dynamometer (Camry Scale) to the nearest kilogram for dominant and nondominant hands. Steps were measured by the vívofit 4 watch (Garmin).

### Statistical Analysis

The study was powered to identify pre- to poststudy changes between the groups. A similar study [60] was used to develop the necessary sample and effect size using the G*power software (version 3.1.9.6; Heinrich Heine University Düsseldorf). Calculations were made for a total sample of 52 participants (26 participants per group) to obtain a statistical difference in muscle strength and mass between the groups, assuming an α of 5%, effect size of 0.72, power of 80%, and 10% inflation for dropouts. Data were imported to SPSS (version 29; IBM Corp) for analysis. DXA measuring muscle and fat mass was the study’s primary outcome measure. Secondary outcomes included protein intake in grams, muscle strength in kilograms, average daily steps, frequency of physical activity in minutes per week, height in inches, and weight in kilograms.

Participants were randomized to the CMG or the CG by block randomization using 2 blocks with 26 codes. On the basis of the assigned participant’s study code, the primary researcher enrolled the participants into their group at the end of their initial visit. Therefore, the allocation was not concealed. The analysis assessed the effect of the intervention with the completers. Any missing data were replaced with the last observation carried forward before analyses of all measurements via single imputation. Participants were excluded from data analysis if they did not complete over 50% of their weekly questionnaires or, after enrollment, met an exclusion criterion.

Results are presented as mean (SD), mean (95% CI), ranges, or frequencies. *P*<.05 was considered statistically significant. Linear mixed models were used to assess the differences in protein intake between the groups at the end of the intervention. The model included the fixed effects of time, intervention, and time-by-intervention interaction. Participants were modeled as a random effect to account for the repeated measures design. When a significant main effect was observed, post hoc analyses were conducted and the Tukey-Kramer method was used to adjust for multiple comparisons. Within-group muscle mass and strength differences, as well as physical activity and diet quality differences, were estimated using an independent samples (1-tailed) *t* test for variables measured before and after the study.

## Results

### Study Population

**Figure 1:**
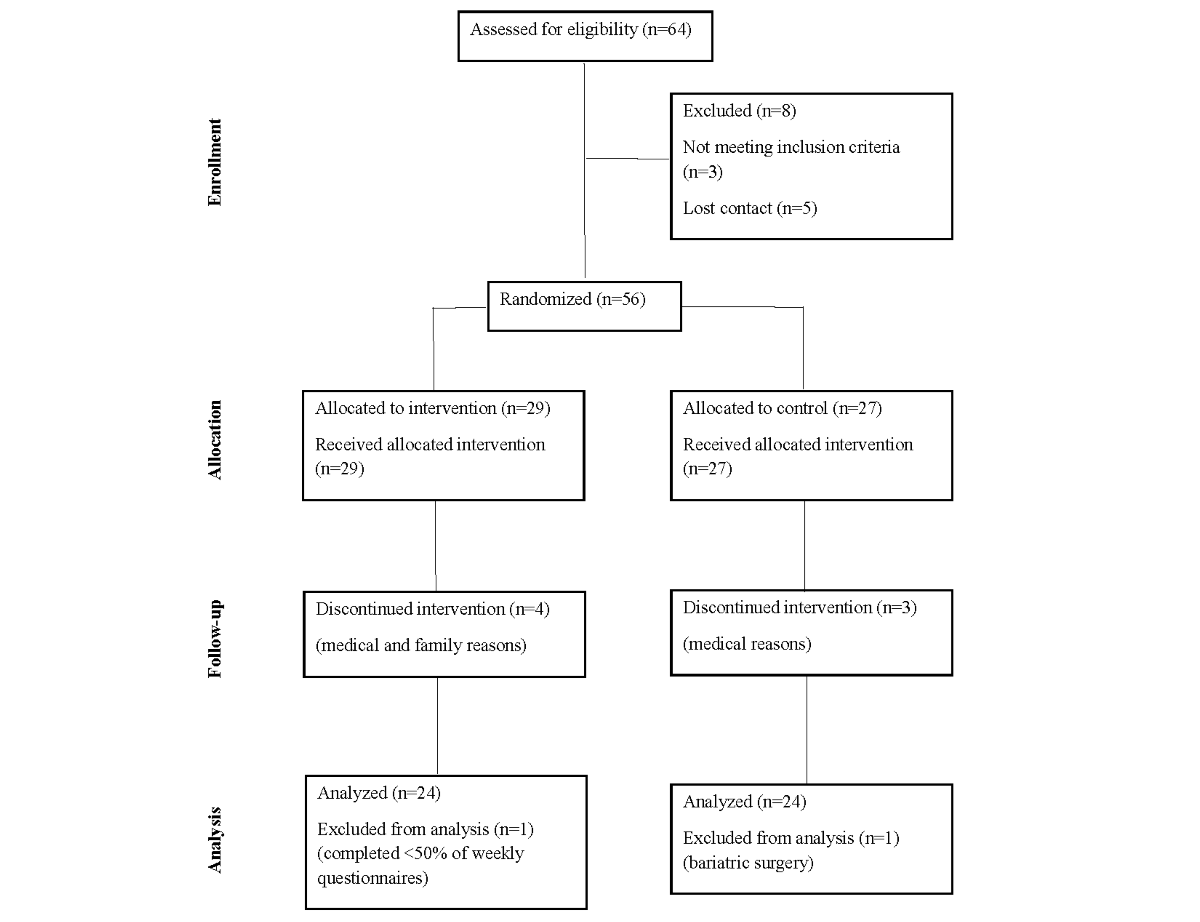
Participants’ CONSORT (Consolidated Standards of Reporting Trials) flow diagram.

In total, 64 participants expressed interest in the study. Of these, 8 (13%) were excluded during web-based or telephone screening due to failing to meet the inclusion criteria or losing contact. A total of 56 participants were eligible for inclusion and were randomized: 29 to the CMG and 27 to the CG. A total of 25 participants in the CMG, compared with 24 in the CG, completed the 16-week weekly questionnaires and both study visits. Of the eligible 56 participants, 7 (13%) withdrew or dropped out before the completion of the study. Of the 7 participants, 6 (86%) dropped out due to medical reasons unrelated to the study, and 1 participant (14%) dropped out due to family reasons. Of the 56 participants, 2 (4%) participants had to be excluded from the data analysis because 1 participant had bariatric surgery during the study and the other completed less than 50% of their weekly questionnaires. Therefore, a total of 49 participants were included for the data analysis (CMG: 24/29, 83%; CG: 23/27, 85%). See the CONSORT (Consolidated Standards of Reporting Trials) study flow diagram (Figure 1) for the study details.

The prestudy characteristics of the groups are presented in Table 1. The study included a greater proportion of female (38/47, 81%) and White (44/47, 94%) participants. The mean age, weight, and BMI of the participants in the CMG were 71.4 (SD 5.2) years, 76.6 (SD 17.4) kg, and 28.0 (SD 6.0) kg/m^2^, respectively. In the CG, they were slightly older (mean 73.2, SD 5.8 years) but had lower weight (mean 69.4, SD 15.0 kg) and BMI (mean 26.1, SD 5.0 kg/m^2^). The CG was found to be more physically active than the CMG. Regarding diet, the CG consumed more fiber, calcium, dairy, vegetables, and fruit than the CMG. Meanwhile, the CMG consumed more daily added sugar than the CG. However, both groups consumed the same amount of daily whole grains.

**Table 1:** Prestudy participant characteristics in body composition, physical activity, and diet components (n=47).

Variable^a^				CMG^b^ (n=24)		CG^c^ (n=23)
**Participant characteristics**						
	**Sex, n (%)**					
		Male	4 (17)		5 (22)	
		Female	20 (83)		18 (78)	
	Age, mean (SD)		71.4 (5.2)		73.2 (5.8)	
	**Age group (years) n (%)**					
		65-74	18 (75)		15 (65)	
		75-84	6 (25)		7 (30)	
		≥85	N/A^d^		1 (4)	
	**Race and ethnicity, n (%)**					
		Black or African American	1 (4)		N/A	
		Hispanic or Mexican American	2 (8)		N/A	
		White	21 (88)		23 (100)	
**Body composition**						
	Weight (kg), mean (SD)		76.6 (17.4)		69.4 (15.0)	
	Height (inches), mean (SD)		65 (3.6)		64.1 (3.7)	
	BMI (kg/m^2^), mean (SD)		28.0 (6.0)		26.1 (5.0)	
	**BMI (kg/m^2^) n (%)**					
		≤18.5, underweight	N/A		2 (9)	
		18.6-24.9, normal	8 (33)		8 (35)	
		25-29.9, overweight	9 (38)		8 (35)	
		30-34.9, class I obesity	4 (17)		5 (22)	
		35-39.9, class II obesity	2 (8)		N/A	
		≥40, class III obesity	1 (4)		N/A	
Physical activity (min/wk), mean (SD)				838.8 (545.9)		930.0 (649.1)
**Diet components, mean (SD)**						
	Fiber (g)		15.9 (2.8)		16.7 (2.9)	
	Calcium (mg)		905.2 (180.6)		932.1 (167.3)	
	Whole grain (ounce)		0.8 (0.3)		0.8 (0.3)	
	Total added sugar (teaspoons)		15.3 (4.9)		13.8 (2.9)	
	Dairy (cup)		1.4 (0.4)		1.6 (0.5)	
	Vegetables (cup)		1.5 (0.3)		1.6 (0.4)	
	Fruit (cup)		0.8 (0.3)		1.0 (0.4)	

^a^Randomized controlled trial (June 2022 to August 2022; Texas Tech University Nutrition and Metabolic Health Initiative) evaluating the effect of a web-based culinary medicine intervention on protein intake, cooking effectiveness, muscle strength, muscle mass, and physical activity in an older adult population aged 65 years and older.

^b^CMG: culinary medicine intervention group.

^c^CG: control group.

^d^N/A: not applicable.

### Muscle Mass and Strength Outcomes

There was no between-group difference in the muscle mass change from the prestudy visit (*P*=.88; Table 2). Using the EWGSOP sarcopenia diagnosis [61], 21% (5/24) of the CMG and 26% (6/23) of the CG had low muscle mass at the prestudy visit. At the poststudy visit, 21% (5/24) of the CMG and 22% (5/23) of the CG had low muscle mass.

**Table 2:** Mean muscle mass and strength of participants at the pre- and poststudy visits (n=47).

Variable^a^			CMG^b^ (n=24), mean (SD)				CG^c^ (n=23), mean (SD)					Poststudy between-group differences, mean (95% CI)			*P* value^d^
			Prestudy		Poststudy	Prestudy			Poststudy						
**Body composition**															
	Muscle mass (g)	40,424.3 (9891.6)		41,042.4 (9857.0)		39,816.9 (7496.0)		39,974.3 (7581.4)		82.9 (−1027.8 to 1193.6)			.88		
	Fat mass (g)	33,043.5 (12,416.6)		33,324.4 (12,654.0)		26,942.5 (9499.2)		26,124.9 (1744.3)		1098.6 (−391.1 to 2588.2)			.14		
	Muscle mass dominant (kg)	20.1 (6.4)		22.0 (6.5)		21.1 (7.5)		22.7 (6.9)		0.1 (−2.4 to 2.7)			.92		
	Muscle mass nondominant (kg)	18.5 (6.4)		20.3 (6.3)		19.2 (7.5)		20.8 (7.9)		0.4 (−1.8 to 2.6)			.72		
	Weight (kg)	76.6 (17.4)		76.9 (17.5)		69.4 (15.0)		68.6 (14.3)		2.6 (−0.7 to 5.9)			.13		
	BMI (kg/m^2^)	28.0 (6.0)		28.0 (6.0)		26.1 (5.0)		25.8 (4.7)		0.3 (−0.3 to 0.8)			.35		
Physical activity (min/wk)			838.8 (545.9)		968.75 (619.9)	930.0 (649.1)			948.3 (602.7)		111.7 (−319.1 to 542.6)			.60	
Steps			—^e^		5921.5 (2887.8)	—			6049.9 (3597.5)		−128.4 (−2040.9 to 1784.1)			.89	

^a^The independent samples *t* test was used to compare between-group differences at the poststudy visit.

^b^CMG: culinary medicine intervention group.

^c^CG: control group.

^d^*P* value refers to between-group differences by the independent samples *t* test.

^e^Not available.

Similar results were seen for muscle strength. There was no between-group difference in the muscle strength change from the prestudy visit (dominant: *P*=.92 and nondominant: *P*=.72). When comparing the classification of muscle strength for the dominant hand, the CMG was considered 29% (7/24) weak, 67% (16/24) normal, and 4% (1/24) strong at the prestudy visit. At the poststudy visit, the CMG was considered 33% (8/24) weak, 46% (11/24) normal, and 21% (5/24) strong. The CG was considered 13% (3/23) weak, 83% (19/23) normal, and 4% (1/23) strong at the prestudy visit. At the poststudy visit, the CG was considered 13% (3/23) weak, 74% (17/23) normal, and 13% (3/23) strong.

When comparing the classification of muscle strength for the nondominant hand, the CMG was considered 42% (10/24) weak, 54% (13/24) normal, and 4% (1/24) strong at the prestudy visit. At the poststudy visit, the CMG was considered 38% (9/24) weak, 50% (12/24) normal, and 13% (3/24) strong. On the other hand, the CG was considered 30% (7/23) weak, 65% (15/23) normal, and 4% (1/23) strong at the prestudy visit. At the poststudy visit, the CG was considered 30% (7/23) weak, 57% (13/23) normal, and 13% (3/23) strong.

Per the EWGSOP sarcopenia diagnosis [61], 38% (9/24) of the CMG and 30% (7/23) of the CG could be diagnosed with probable sarcopenia. In comparison, 8% (2/24) of the CMG and 9% (2/23) of the CG could be diagnosed with sarcopenia at the prestudy visit. At the poststudy visit, 33% (8/24) of the CMG and 17% (4/23) of the CG could be diagnosed with probable sarcopenia, whereas 8% (2/24) of the CMG and 9% (2/23) of the CG could be diagnosed with sarcopenia at the poststudy visit.

### Protein Intake and Diet Quality

**Figure 2:**
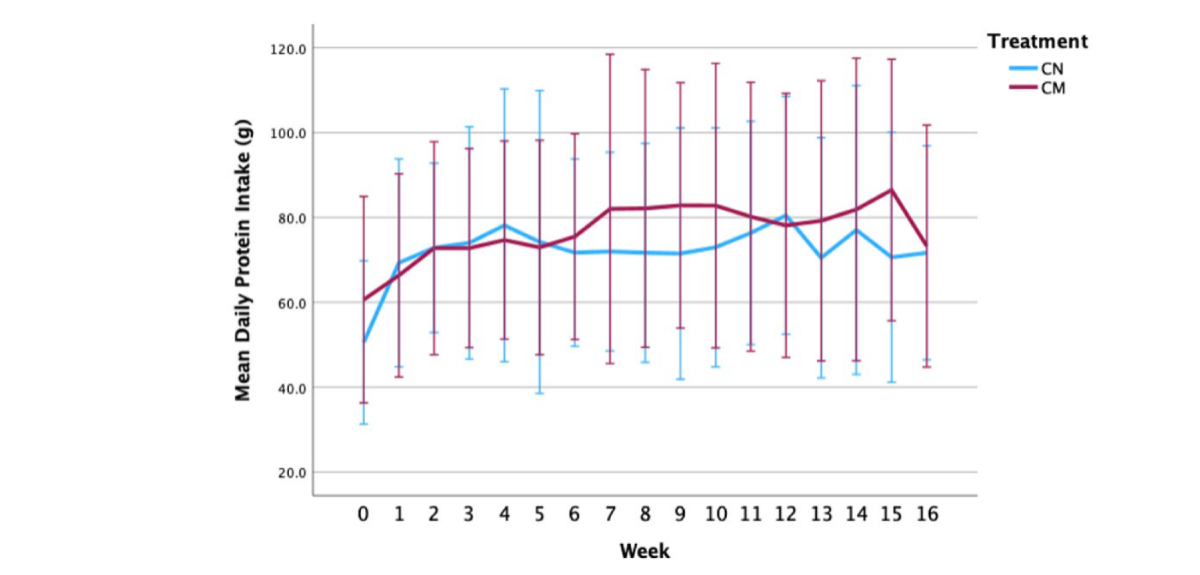
Mean (SD) daily protein intake in grams for each week of the study for each group. CM: culinary medicine; CN: control.

Figure 2 reveals the mean (SD) daily protein intake in grams for each week of the study for each group. A nonsignificant time-by-intervention interaction was observed for daily protein intake (Figure 2 and Table 3; *P*=.08). There was also no statistically significant difference in protein intake between the interventions (*P*=.50). However, a statistically significant time effect was observed (*P*≤.001). Post hoc testing showed that daily protein intake was significantly higher at weeks 1 to 16 versus week 0 (*P*<.05) in the cohort. At week 16, protein intake was 16.9 (95% CI 5.77-27.97) g higher than that at the prestudy visit.

**Table 3:** Dietary intake of participants at the pre- and poststudy visits (N=47).

Variable^a^	CMG^b^ (n=24), mean (SD)		CG^c^ (n=23), mean (SD)		Poststudy between-group differences, mean (95% CI)	*P* value^d^
	Prestudy	Poststudy	Prestudy	Poststudy		
Protein (g)	60.6 (5.1)	73.3 (5.4)	50.5 (5.2)	71.7 (5.4)	−8.5 (−22.6 to 5.6)	.08
Fiber (g)	15.9 (2.8)	16.3 (2.5)	16.7 (2.9)	16.1 (2.8)	1.1 (−0.9 to 3.0)	.29
Calcium (mg)	905.2 (180.6)	904.4 (164.6)	932.1 (167.3)	930.5 (251.3)	0.8 (−102.5 to 104.1)	.99
Whole grain (ounce)	0.8 (0.3)	0.7 (0.3)	0.8 (0.3)	0.7 (0.3)	−0.01 (−0.3 to 0.2)	.93
Total added sugar (teaspoons)	15.3 (4.9)	12.5 (2.7)	13.8 (2.9)	13.2 (2.3)	−2.3 (−4.8 to 0.2)	.08
Dairy (cup)	1.4 (0.4)	1.4 (0.4)	1.6 (0.5)	1.6 (0.7)	−0.01 (−0.3 to 0.3)	.94
Vegetables (cup)	1.5 (0.3)	1.6 (0.4)	1.6 (0.4)	1.6 (0.4)	0.1 (−0.2 to 0.4)	.49
Fruit (cup)	0.8 (0.3)	0.8 (0.3)	1.0 (0.4)	0.8 (0.2)	0.2 (−0.02 to 0.5)	.07

^a^Linear mixed-effects model analysis was used to compare between-group differences after the study for protein, whereas an independent samples *t* test was used for the remaining variables.

^b^CMG: culinary medicine intervention group.

^c^CG: control group.

^d^*P* value refers to linear mixed-effects model analysis of between-group differences over time (time×treatment interaction).

Each group was evaluated to see how many participants met their protein needs (1.0-1.2 g/kg body mass per day). In the CG, 39% (9/23) participants did not meet their needs, 26% (6/23) did meet their needs, and 35% (8/23) exceeded their needs during the study. In the CMG, 58% (14/24) participants did not meet their needs, 8% (2/24) did meet their needs, and 33% (8/24) exceeded their needs during the study. Additionally, in all the completed protein questionnaires, the CMG and the CG had blank answers for 15.4% (63/408) and 12.5% (49/391) of their questions, respectively . When evaluating the daily intake for each dietary component from the DSQ (Table 3), the components stayed close to the same when comparing pre- with poststudy results.

### Cooking Effectiveness

For the CMG, participants reported watching 82.8% (318/384) of the intervention videos. The primary reason reported on why they did not watch the videos was “not interested in watching” (21/56, 38%). Additional reasons included personal reasons, traveling or vacation, or they did not receive the video. For the CG, participants reported that they read 94.8% (349/368) of the recipes sent to them. The primary reason why the participants did not read the recipe was “busy” (5/13, 39%). Additional reasons included personal and medical reasons, laziness, uninterest, not receiving the video, and having their spouse read it.

When examining whether both groups cooked the recipe learned through web-based videos or just by reading the recipe, the CMG cooked more recipes than the CG (64.8%, 249/384, vs 62.5%, 230/368). Based on the questionnaires with responses outside of “N/A,” the CMG and CG did not cook primarily because of “holiday, traveling, or vacation” (CMG: 20%, 25/125, and CG: 26.5%, 35/132). See Table 4 for the remaining reasons. Barriers or complications that were reported from both groups when either watching the videos or preparing the recipe included borrowing ingredients from a neighbor; recipe serving size being too big; confusion toward either the ingredients or methods; changing or not including ingredients to meet taste or diet preference; finding certain ingredients at the store; too much spice or ingredient in the recipe for their palette; standing for an extended period was challenging; difficulties in scheduling time and energy to shop, prepare, or cook; or taking more initiative to prepare recipe themselves.

**Table 4:** Reasons for not cooking during the study.

Reasons	CMG^a^ (n=125), n (%)	CG^b^ (n=132), n (%)
Holiday, traveling, or vacation	25 (20)	35 (26.5)
Busy	16 (12.8)	26 (19.7)
Spouse prepared it	1 (0.8)	17 (12.9)
Not interested in cooking	18 (14.4)	14 (10.6)
Ate leftovers	1 (0.8)	11 (8.3)
Medical reason	9 (7.2)	7 (5.3)
Fixed other recipe	17 (13.6)	6 (4.5)
Did not go to the store	5 (4)	4 (3)
Recipe too large	3 (2.4)	4 (3)
Food preference	19 (15.2)	3 (2.3)
Ate out	1 (0.8)	2 (1.5)
Did not have the recipe	0 (0)	1 (0.8)
Financial reason	0 (0)	1 (0.8)
No reason provided	2 (1.6)	1 (0.8)
Personal reason	6 (4.6)	0 (0)
Confusion toward ingredients	2 (1.6)	0 (0)

^a^CMG: culinary medicine intervention group.

^b^CG: control group.

At the end of the study, both groups were asked about the main challenges or barriers to maintaining their protein intake (Tables 5 and 6). Meanwhile, the CMG participants were asked how the CM videos specifically helped clarify managing their protein intake (Table 7) and what the most memorable thing they recalled after watching the video or what their favorite part of the CM videos was. All CMG participants were reported having no technical difficulties accessing and watching the videos.

**Table 5:** Culinary medicine intervention group’s main challenges or barriers to maintaining their protein intake.

Challenge or barrier	Value (n=24), n (%)
Finding low-fat protein options	1 (4)
I was eating all the time	1 (4)
Keeping track of protein intake	1 (4)
Knowing which protein is healthiest or easiest	1 (4)
Limiting protein intake	1 (4)
Not consuming enough daily	1 (4)
Price of protein	1 (4)
Deciding the correct protein amount to eat	2 (8)
Protein variety	3 (13)
No answer provided	3 (13)
No issues	9 (38)

**Table 6:** Control group’s main challenges or barriers to maintaining their protein intake.

Challenge or barrier	Value (n=23), n (%)
High calories in cheese or red meat	1 (4)
Paying attention when shopping	1 (4)
Time to prepare	1 (4)
Eating 25-30 g of protein was too much for me	1 (4)
No answer provided	3 (13)
No issue	16 (70)

**Table 7:** How the culinary medicine videos specifically helped clarify managing their protein intake.

Reason	Value (n=24), n (%)
Understanding serving or portion size	7 (29)
New cooking ideas	2 (8)
How important protein is to our health	2 (8)
I am visual learner, so helped my confidence	2 (8)
I realized that I do not eat enough protein	2 (8)
Introduce more protein into my own recipes	1 (4)
Learning new skills in cooking	1 (4)
How easy it is to manage protein intake by cooking myself	1 (4)
Hard to tell how much protein I got from eating a serving size	1 (4)
Helped but I get busy and forget to eat during the day	1 (4)
Waste of time	1 (4)
No answers provided	3 (13)

## Discussion

### Principal Findings

To the authors’ knowledge, a study has yet to be performed with CM explicitly targeting the older adult population to enhance their protein intake. However, a statistically significant time effect was observed (*P*≤.001). Furthermore, post hoc testing showed that daily protein intake was significantly higher at weeks 1 to 16 versus week 0 (*P*<.05). At week 16, protein intake was 16.9 (95% CI 5.77-27.97) g higher than that at the prestudy visit. This result indicates that protein intake increased in the cohort with the information provided to both groups.

Nevertheless, there was no additive effect of the CMG over what the CG received because no between-group differences were observed for any primary or secondary outcomes. Insufficient consistent protein intake, low physical activity, adherence to the intervention, and accuracy of the questionnaires could explain the results. Also, participants’ ethnicity, average age, gender, and BMI were similar in both groups and affected the diversity of the study’s population; therefore, the outcomes were not tested against them because there was no vast difference to indicate a relationship. Given the limited representation of men in the cohort, the results cannot be generalized to men, Hispanic participants, and African American participants.

The accuracy of each group’s protein questionnaire could play a factor because they were self-administered. Self-administered questionnaires are more susceptible to item nonresponse [62]. The CMG and the CG had blank answers for 15.4% (63/408) and 12.5% (49/391) of their questions, respectively, suggesting that their intake could have been higher and explained how their muscle mass was overall maintained. Additionally, the participants were not asked to change their diet outside their protein intake. The DSQ reported that participants’ diets did remain the same.

### Comparison With Prior Work

Before the study started, both groups were recommended to consume 25 to 30 g of protein per meal in addition to daily physical activity. These recommendations are similar to Paddon-Jones and Rasmussen’s [63] findings, reporting that approximately 25 to 30 g of protein per meal is a valuable strategy for maintaining muscle mass in older adults. This strategy would mean that the participants would have to eat approximately 75 to 90 g of protein daily. The CMG met this range from weeks 6 to 15, but the CG met this range during weeks 4, 11, and 12. Specifically, 39% (9/23) of the CG and 58% (14/24) of the CMG did not meet their needs (1.0-1.2 g/kg body weight per day). The 2005-2014 NHANES [26] reported that 31% to 50% of older adults did not meet their protein recommendations. Our population was in this range. Therefore, these results could also explain why muscle mass did not significantly increase between the groups. However, the estimated average requirement for 51 to 70 years is 0.66 g/kg/d, and the recommended dietary intake is 0.8 g/kg/d for all adults over 18 years old, including older adults [64]. Therefore, in the context of adequate energy intake, muscle mass was maintained in this cohort if their protein intake was consistent with these levels.

Grip strength has been used in research to determine overall body strength [65,66]. However, there were no between-group differences in muscle strength change from the prestudy visit. Kim et al [67] found no association between the amount and change (increase or decrease) in daily total protein intake with the incidence or prevention of low muscle strength, which was similar to our results. Additionally, a longitudinal study [68] indicated that 25 to 30 g of protein per meal is associated with greater muscle strength in older adults. However, this recommended intake did not consistently happen in our study, and participants did not meet their calculated needs, which could affect their muscle strength. Physical activity also did not impact muscle strength. Similar results were seen with Ramsey et al [69], who also saw no association between the number of steps and handgrip strength.

When looking at their steps, current evidence suggests that healthy older adults should meet approximately 7000 to 10,000 steps per day [70]. However, our study showed that 67% (16/24) of the CMG and 57% (13/23) of the CG did not meet this range. Also, Park et al [34] reported that individuals who walked at least 7000 to 8000 steps daily likely have muscle mass above the sarcopenia threshold. Because only 33% of the CMG and 44% of the CG met this threshold, it is unsurprising that their lack of steps may have impacted our results.

Lastly, the dropout rates were similar, 14% (4/29) in the CMG and 11% (3/27) in the CG. This rate is lower than the reported average of 20% to 49%, which is commonly seen in dietary clinical trials [71]. In the CMG, 10% (3/29) of participants dropped out due to medical reasons, whereas 3% (1/29) dropped out due to family reasons. In the CG, all the participants dropped out due to medical reasons. These are all common reasons for dropouts in clinical trials [72]. The dropouts were not related to the study, and no adverse effects were reported throughout the study.

### Strengths and Limitations

#### Strength

This study is the first to evaluate CM’s effect on enhancing protein intake and muscle quality in older adults, which brings a new aspect to existing CM research. Furthermore, this study allowed us to see if the intervention program improved their knowledge, awareness, and attitude toward protein intake within 4 months. In addition, the feedback from the participants can be applied to future studies.

A registered dietitian (RD), fully trained and qualified with years of experience, developed the whole program with assistance from those with expertise in food service and kinesiology. In addition, an RD implemented the intervention and provided advice if participants needed clarification about their intervention.

Our study had an overall dropout rate and data exclusion of 16% (9/56), limiting attrition bias. Additionally, there was a high response rate to the weekly questionnaires, with 84.6% (345/408) for the CMG versus 87.5% (342/391) for the CG, and the response rate goal for most research was approximately 60% [73]. This high response rate was credited to weekly adherence checks and effective accountability in recording their weekly questionnaires. Lastly, this intervention was low-cost and could be easily replicated and enhanced for future research.

#### Limitations

Although exercise recommendation handouts were given in this study, the main intervention has limitations with a focus on diet and nutrition education. A more comprehensive approach including digital CM education, exercise training sessions, and dietary supplementation would have allowed for a more adequate comparison and expectation of significant differences in muscle quality outcomes. Additionally, the result of this study may not be representative of the general population because the majority were female (38/47, 81%) and White (44/47, 94%), and their ages were similar. Therefore, this study would benefit from seeing its effect on those who lack cooking confidence and skills in addition to a more diverse population setting. In addition, there may be recall and social desirability biases as the questionnaires were self-reported, and the participants knew that the research team was reading the responses. This factor could be lessened through the interview-administered questionnaires. Finally, the protein questionnaire results may not be accurate because of the blank questions.

Some participants reported that they could not cook a recipe because they were on Weight Watchers or had self-proclaimed dietary restrictions (eg, no bread or pasta). This situation was seen in 15.2% (19/125) of the CMG and 2.3% (3/132) of the CG. Also, participants reported that some recipes could have been better for a different season (eg, chili in the winter instead of during the summer). They also voiced concern about some recipes needing smaller portions because they live alone. Additionally, because this intervention was performed in summer, fall, and the beginning of winter, the seasonal changes can explain why participants did not partake in some weeks of the study. For example, the participants did not cook their recipes because of holidays, traveling, or vacations (CMG: 20%, 25/125, and CG 26.5%, 35/132). Another example is that the colder weather and traveling could have impacted the results of the steps because most of the questions asked were about outdoor and in-house activities.

### Conclusions and Future Direction

To the authors’ knowledge, this study is the first to examine the outcomes of CM in the form of web-based cooking demonstrations and nutrition education to enhance protein intake and muscle quality in older adults. The results reveal insufficient evidence because no between-group differences were observed for primary or secondary outcomes. However, most of the intervention group reported that the cooking demonstrations helped them prepare and cook recipes at home, providing more confidence in the kitchen, and its learning was feasible for them.

In the future, it would be valuable to further investigate the factors that could have affected this study. In developing and implementing this study, exercise training sessions and a dietary supplement could be included. Additionally, the research study design could include RDs, chefs, exercise physiologists, health coaches, or psychologists. The staff would be essential in creating the study protocol, kitchen equipment checklist, consent forms, scripts, and questions. During recruitment, it would be ideal to obtain a broad age range with an equal gender and ethnicity ratio to help reciprocate the general population. The recipes should consider the season, 1-person portion size, time, cost, and mild flavors. A protein food diary could help keep track of protein intake during the week and help answer the protein questionnaire accurately.

It could be interesting to incorporate muscle biopsy and biomarkers, such as vitamin B_12_, folate, and creatinine, to evaluate muscle mass further and see if this intervention impacts or could explain why muscle mass outcomes were nonsignificant due to predispositions. However, there are challenges in successfully performing a muscle biopsy in older men and women who are frail or have low body mass [74], so that would be a concern to consider. For biomarkers, no specific recommendations, references, or cutoff values are available to assess muscle mass or quality. Therefore, the biomarkers could be used to notice any significant change within a short time duration. Overall, given the current concern of sarcopenia, these concepts could enhance this intervention further with the information gathered in this study to impact public health.

## Appendix

Multimedia Appendix 1 CONSORT eHEALTH Checklist (V 1.6.2).

## Abbreviations

CG: control group
CHAMPS: Community Healthy Activities Model Program for Seniors
CM: culinary medicine
CMG: culinary medicine intervention group
CONSORT: Consolidated Standards of Reporting Trials
DSQ: Dietary Screener Questionnaire
DXA: dual-energy x-ray absorptiometry
EWGSOP: European Working Group on Sarcopenia in Older People
MPS: muscle protein synthesis
NHANES: National Health and Nutrition Examination Survey
NMHI: Nutrition and Metabolic Health Initiative
RD: registered dietitian
SMM: skeletal muscle mass

## Data Availability

The data sets generated and analyzed during this study are available from the corresponding author on reasonable request.
