# Supplementary material for: The Effect of Web-Based Culinary Medicine to Enhance Protein Intake on Muscle Quality in Older Adults: Randomized Controlled Trial
Source: JMIR Form Res. 2024 Feb 13;8:e49322. doi: 10.2196/49322 (PMC10900082; doi:10.2196/49322)
Supplement: Multimedia Appendix 1 [file formative_v8i1e49322_app1.pdf]

CONSORT-EHEALTH Checklist V1.6.2 Report	Manuscript Number	49322
**(based on CONSORT-EHEALTH V1.6), available at [http://tinyurl.com/consort-ehealth-v1-6].**		
**Date completed**		
2/9/2024 15:17:32		
**by**		
Shannon Galyean		
The Effect of Web-Based Culinary Medicine to Enhance Protein Intake on Muscle Quality in Older Adults: Randomized Controlled Trial		
TITLE		
**1a-i) Identify the mode of delivery in the title**		
The delivery in the title is "Web-Based"		
**1a-ii) Non-web-based components or important co-interventions in title**		
**1a-iii) Primary condition or target group in the title**		
The Effect of Web-Based Culinary Medicine to Enhance Protein Intake on Muscle Quality in Older Adults: Randomized Controlled Trial		
ABSTRACT		
**1b-i) Key features/functionalities/components of the intervention and comparator in the METHODS section of the ABSTRACT**		
The intervention group received weekly web-based cooking demonstrations and biweekly nutrition education videos about enhancing protein intake		
**1b-ii) Level of human involvement in the METHODS section of the ABSTRACT**		
**1b-iii) Open vs. closed, web-based (self-assessment) vs. face-to-face assessments in the METHODS section of the ABSTRACT**		
A closed online user group was provided the intervention videos.		
**1b-iv) RESULTS section in abstract must contain use data**		
**1b-v) CONCLUSIONS/DISCUSSION in abstract for negative trials**		
INTRODUCTION		
**2a-i) Problem and the type of system/solution**		
"Aging can lead to age-related musculoskeletal disorders"; "The protein quality is also critical in age-related skeletal muscle mass (SMM) anabolism."; 'Culinary medicine (CM) is a novel evidence-based medical field defined by combining the science of medicine with food and cooking; "A web-based CM program might be an innovative strategy to improve protein intake in independent older adults at home."		
**2a-ii) Scientific background, rationale: What is known about the (type of) system**		

"More interventions focusing on nutrition and lifestyle changes are essential in decreasing chronic disease and health care costs [45]. Educating and empowering individuals to change their lifestyles can be less costly than medications and invasive procedures [45]. Culinary medicine (CM) is a novel evidence-based medical field defined by combining the science of medicine with food and cooking [46]. CM differs from traditional lifestyle and nutrition interventions by attempting to empower the patient to care for herself or himself safely, effectively, and happily with food and beverages as a primary care technique [47]. It helps people access and eat nutrient-dense meals to prevent and treat potential chronic diseases [46]. Individuals learn and practice culinary skills while tasting new recipes [45]. Also, they can incorporate their favorite foods into their eating plan while learning how to enhance diet quality through new foods (eg, different types of vegetables) and meal preparation tips (eg, defrosting techniques) [47,48]. If executed appropriately, CM can be taught to all populations regardless of culinary skill, educational level, or socioeconomic background [45]. A CM curriculum typically includes practical applications in supermarkets and home kitchens [49]. These practical applications include basic nutrition knowledge and instruction on how to apply that knowledge to diet therapies [49]. However, limited studies report whether a web-based CM curriculum could be as effective as in-person.<br>Multiple randomized controlled trials report that CM significantly improved individuals' culinary knowledge, healthy dietary patterns, and self-efficacy for healthier cooking [50-54]. Thus, highlighting CM's potential as a nutrition intervention could lower the risk of diet-related chronic disease among older adults. However, few studies in this area include older adult participants; none exclusively focused on an older adult population, and only 6% of CM programs were taught by a qualified health professional [55]. Additionally, CM interventions have been very heterogeneous, indicating a lack of variety in how the intervention is conducted compared with others [55]. Therefore, this study could advance our knowledge of CM and sarcopenia prevention in older adults. A web-based CM program might be an innovative strategy to improve protein intake in independent older adults at home. In addition, this program could successfully reduce barriers to protein intake, enabling older adults to enhance their diet and muscle quality. This factor could be vital because research surrounding CM within older adults is in its infancy. Therefore, our study aimed to examine how a web-based CM intervention, emphasizing convenient ways to increase lean red meat intake, could improve protein intake with the promotion of physical activity to see how this intervention could affect older adults' muscle strength and mass."		
**Does your paper address CONSORT subitem 2b?**		
"CM differs from traditional lifestyle and nutrition interventions by attempting to empower the patient to care for herself or himself safely, effectively, and happily with food and beverages as a primary care technique. It helps people access and eat nutrient-dense meals to prevent and treat potential chronic diseases. Individuals learn and practice culinary skills while tasting new recipes. Also, they can incorporate their favorite foods into their eating plan while learning how to enhance diet quality through new foods (eg, different types of vegetables) and meal preparation tips (eg, defrosting techniques). If executed appropriately, CM can be taught to all populations regardless of culinary skill, educational level, or socioeconomic background. A CM curriculum typically includes practical applications in supermarkets and home kitchens. These practical applications include basic nutrition knowledge and instruction on how to apply that knowledge to diet therapies. However, limited studies report whether a web-based CM curriculum could be as effective as in-person.<br>Multiple randomized controlled trials report that CM significantly improved individuals' culinary knowledge, healthy dietary patterns, and self-efficacy for healthier cooking. Thus, highlighting CM's potential as a nutrition intervention could lower the risk of diet-related chronic disease among older adults. However, few studies in this area include older adult participants; none exclusively focused on an older adult population, and only 6% of CM programs were taught by a qualified health professional. Additionally, CM interventions have been very heterogeneous, indicating a lack of variety in how the intervention is conducted compared with others. Therefore, this study could advance our knowledge of CM and sarcopenia prevention in older adults. A web-based CM program might be an innovative strategy to improve protein intake in independent older adults at home. In addition, this program could successfully reduce barriers to protein intake, enabling older adults to enhance their diet and muscle quality. "		
METHODS		
**3a) CONSORT: Description of trial design (such as parallel, factorial) including allocation ratio**		
"A 16-week single-center, parallel-group, randomized study compared a web-based CM intervention group (CMG) with a control group (CN) on their protein intake, cooking effectiveness, muscle strength, muscle mass, and physical activity."		
**3b) CONSORT: Important changes to methods after trial commencement (such as eligibility criteria), with reasons**		
There were no content changes.		
**3b-i) Bug fixes, Downtimes, Content Changes**		
**4a) CONSORT: Eligibility criteria for participants**		

"The inclusion criteria involved individuals aged 65 years or older, able to cook for themselves, physically active (eg, no need for equipment for assistance), and able to use a computer and mobile device. The exclusion criteria included individuals aged <65 years, with limited mobility (eg, need for equipment for assistance), cognitive dysfunction (eg, dementia), heart pacemaker, type 1 or type 2 diabetes with insulin use, current smokers, some form of amputation, unable to use a computer and mobile device, unable or unwilling to wear the vívofit 4 watch (Garmin) for the duration of the study, and those undergoing or had recently undergone a severe medical procedure or diagnosis."		
**4a-i) Computer / Internet literacy**		
**4a-ii) Open vs. closed, web-based vs. face-to-face assessments:**		
Participants were recruited at senior centers face-to-face. The intervention was delivered online from closed access you tube videos. pre and post intervention appointments were face-to-face.		
**4a-iii) Information giving during recruitment**		
**4b) CONSORT: Settings and locations where the data were collected**		
"Weekly activity level was measured through CHAMPS, the diet by a DSQ, protein intake through a protein questionnaire, cooking confidence and attitude using a pre- and poststudy cooking effectiveness questionnaire, and intervention compliance through weekly cooking effectiveness." These were measured by online database. "grip strength, height, and weight were measured. Then, the participants were scanned by dual-energy x-ray absorptiometry (DXA)." These were measured face-to-face.		
**4b-i) Report if outcomes were (self-)assessed through online questionnaires**		
"Weekly activity level was measured through CHAMPS, the diet by a DSQ, protein intake through a protein questionnaire, cooking confidence and attitude using a pre- and poststudy cooking effectiveness questionnaire, and intervention compliance through weekly cooking effectiveness." These were measured by online database. "grip strength, height, and weight were measured. Then, the participants were scanned by dual-energy x-ray absorptiometry (DXA)." These were measured face-to-face.		
**4b-ii) Report how institutional affiliations are displayed**		
**5) CONSORT: Describe the interventions for each group with sufficient details to allow replication, including how and when they were actually administered**		
**5-i) Mention names, credential, affiliations of the developers, sponsors, and owners**		
**5-ii) Describe the history/development process**		
**5-iii) Revisions and updating**		
**5-iv) Quality assurance methods**		
**5-v) Ensure replicability by publishing the source code, and/or providing screenshots/screen-capture video, and/or providing flowcharts of the algorithms used**		
**5-vi) Digital preservation**		
**5-vii) Access**		
The participants received youtube videos that contained the content of the intervention via email.		
**5-viii) Mode of delivery, features/functionalities/components of the intervention and comparator, and the theoretical framework**		
the intervention included cooking demos and nutrition education videos that included culinary medicine.		
**5-ix) Describe use parameters**		
**5-x) Clarify the level of human involvement**		
**5-xi) Report any prompts/reminders used**		

The participants were reminded via email if they didn't complete their questionnaires.		
**5-xii) Describe any co-interventions (incl. training/support)**		
"The CMG received weekly web-based cooking demonstrations with a recipe handout and biweekly nutrition education video on general nutrition information based on the Nutrition Care Manual content from the Academy of Nutrition and Dietetics [56], all provided by email at the beginning of each week. Meanwhile, the CNG just received the recipe handout by email."		
**6a) CONSORT: Completely defined pre-specified primary and secondary outcome measures, including how and when they were assessed**		
"Community Healthy Activities Model Program for Seniors (CHAMPS), Dietary Screener Questionnaire (DSQ), protein, and cooking effectiveness." The first two were validated questionnaires. The last two were to assess the effectiveness of the intervention.		
**6a-i) Online questionnaires: describe if they were validated for online use and apply CHERRIES items to describe how the questionnaires were designed/deployed**		
**6a-ii) Describe whether and how "use" (including intensity of use/dosage) was defined/measured/monitored**		
**6a-iii) Describe whether, how, and when qualitative feedback from participants was obtained**		
**6b) CONSORT: Any changes to trial outcomes after the trial commenced, with reasons**		
"Weekly activity level was measured through CHAMPS, the diet by a DSQ, protein intake through a protein questionnaire, cooking confidence and attitude using a pre- and poststudy cooking effectiveness questionnaire, and intervention compliance through weekly cooking effectiveness." These were measured by online database. "grip strength, height, and weight were measured. Then, the participants were scanned by dual-energy x-ray absorptiometry (DXA)." These were measured face-to-face.		
**7a) CONSORT: How sample size was determined**		
**7a-i) Describe whether and how expected attrition was taken into account when calculating the sample size**		
**7b) CONSORT: When applicable, explanation of any interim analyses and stopping guidelines**		
"Community Healthy Activities Model Program for Seniors (CHAMPS), Dietary Screener Questionnaire (DSQ), protein, and cooking effectiveness." The first two were validated questionnaires. The last two were to assess the effectiveness of the intervention.		
**8a) CONSORT: Method used to generate the random allocation sequence**		
"Participants were randomized to the CMG or the CNG by block randomization using 2 blocks with 26 codes. On the basis of the assigned participant's study code, the primary researcher enrolled the participants into their group at the end of their initial visit. Therefore, the allocation was not concealed."		
**8b) CONSORT: Type of randomisation; details of any restriction (such as blocking and block size)**		
"Participants were randomized to the CMG or the CNG by block randomization using 2 blocks with 26 codes."		
**9) CONSORT: Mechanism used to implement the random allocation sequence (such as sequentially numbered containers), describing any steps taken to conceal the sequence until interventions were assigned**		
"Participants were randomized to the CMG or the CNG by block randomization using 2 blocks with 26 codes."		
**10) CONSORT: Who generated the random allocation sequence, who enrolled participants, and who assigned participants to interventions**		
The manuscript does not specifiy this subitem.		
**11a) CONSORT: Blinding - If done, who was blinded after assignment to interventions (for example, participants, care providers, those assessing outcomes) and how**		
**11a-i) Specify who was blinded, and who wasn't**		
There was no blinding in this study.		
**11a-ii) Discuss e.g., whether participants knew which intervention was the "intervention of interest" and which one was the "comparator"**		
**11b) CONSORT: If relevant, description of the similarity of interventions**		
There was no blinding in this study.		
**12a) CONSORT: Statistical methods used to compare groups for primary and secondary outcomes**		
"Our study had an overall dropout rate and data exclusion of 16% (9/56), limiting attrition bias. Additionally, there was a high response rate to the weekly questionnaires, with 84.6% (345/408) for the CMG versus 87.5% (342/391) for the CNG, and the response rate goal for most research was approximately 60% "		
**12a-i) Imputation techniques to deal with attrition / missing values**		

"The analysis assessed the effect of the intervention with the completers. Any missing data were replaced with the last observation carried forward before analyses of all measurements via single imputation."		
**12b) CONSORT: Methods for additional analyses, such as subgroup analyses and adjusted analyses**		
There were no subgroup analyses.		
RESULTS		
**13a) CONSORT: For each group, the numbers of participants who were randomly assigned, received intended treatment, and were analysed for the primary outcome**		
"A total of 56 participants were eligible for inclusion and were randomized: 29 to the CMG and 27 to the CNG. A total of 25 participants in the CMG, compared with 24 in the CNG, completed the 16-week weekly questionnaires and both study visits."		
**13b) CONSORT: For each group, losses and exclusions after randomisation, together with reasons**		
There is a CONSORT flow diagram included as part of the manuscript.		
**13b-i) Attrition diagram**		
**14a) CONSORT: Dates defining the periods of recruitment and follow-up**		
"Participants were recruited and enrolled from June 2022 to August 2022, with data collection completed in December 2022. "		
**14a-i) Indicate if critical "secular events" fell into the study period**		
**14b) CONSORT: Why the trial ended or was stopped (early)**		
The trial ended as intended as we completed recruitment, participants completed the study requirements, and data was collected.		
**15) CONSORT: A table showing baseline demographic and clinical characteristics for each group**		
There is a table showing demographic information.		
**15-i) Report demographics associated with digital divide issues**		
Our study reported age, gender, and ethnicity.		
**16a) CONSORT: For each group, number of participants (denominator) included in each analysis and whether the analysis was by original assigned groups**		
**16-i) Report multiple "denominators" and provide definitions**		
We report mulitple denominators and provide definition.		
**16-ii) Primary analysis should be intent-to-treat**		
**17a) CONSORT: For each primary and secondary outcome, results for each group, and the estimated effect size and its precision (such as 95% confidence interval)**		
For all outcomes, we provide the p-value.		
**17a-i) Presentation of process outcomes such as metrics of use and intensity of use**		
**17b) CONSORT: For binary outcomes, presentation of both absolute and relative effect sizes is recommended**		
For binary outcomes, we provide the absolute value and percentage.		
**18) CONSORT: Results of any other analyses performed, including subgroup analyses and adjusted analyses, distinguishing pre-specified from exploratory**		
We do not report any subgroup analyses.		
**18-i) Subgroup analysis of comparing only users**		
**19) CONSORT: All important harms or unintended effects in each group**		
We did not have any harms or unintended effects to report.		
**19-i) Include privacy breaches, technical problems**		
**19-ii) Include qualitative feedback from participants or observations from staff/researchers**		
DISCUSSION		

**20) CONSORT: Trial limitations, addressing sources of potential bias, imprecision, multiplicity of analyses**		
**20-i) Typical limitations in ehealth trials**		
"Although exercise recommendation handouts were given in this study, the main intervention has limitations with a focus on diet and nutrition education. A more comprehensive approach including digital CM education, exercise training sessions, and dietary supplementation would have allowed for a more adequate comparison and expectation of significant differences in muscle quality outcomes. Additionally, the result of this study may not be representative of the general population because the majority were female (38/47, 81%) and Caucasian (44/47, 94%), and their age was similar. Therefore, this study would benefit from seeing its effect on those who lack cooking confidence and skills in addition to a more diverse population setting. In addition, there may be recall and social desirability biases as the questionnaires were self-reported, and the participants knew that the research team was reading the responses. This factor could be lessened through the interview-administered questionnaires. Finally, the protein questionnaire results may not be accurate because of the blank questions.<br>Some participants reported that they could not cook a recipe because they were on Weight Watchers or had self-proclaimed dietary restrictions (eg, no bread or pasta). This situation was seen in 15.2% (19/125) of the CMG and 2.3% (3/132) of the CNG. Also, participants reported that some recipes could have been better for a different season (eg, chili in the winter instead of during the summer). They also voiced concern about some recipes needing smaller portions because they live alone. Additionally, because this intervention was performed in summer, fall, and the beginning of winter, the seasonal changes can explain why participants did not partake in some weeks of the study. For example, the participants did not cook their recipes because of holidays, traveling, or vacations (CMG: 20%, 25/125, and CNG 26.5%, 35/132). Another example is that the colder weather and traveling could have impacted the results of the steps because most of the questions asked were about outdoor and in-house activities."		
**21) CONSORT: Generalisability (external validity, applicability) of the trial findings**		
**21-i) Generalizability to other populations**		
**21-ii) Discuss if there were elements in the RCT that would be different in a routine application setting**		
**22) CONSORT: Interpretation consistent with results, balancing benefits and harms, and considering other relevant evidence**		
**22-i) Restate study questions and summarize the answers suggested by the data, starting with primary outcomes and process outcomes (use)**		
"this study is the first to examine the outcomes of CM in the form of web-based cooking demonstrations and nutrition education to enhance protein intake and muscle quality in older adults. The results reveal insufficient evidence because no between-group differences were observed for primary or secondary outcomes. However, most of the intervention group reported that the cooking demonstrations helped them prepare and cook recipes at home, providing more confidence in the kitchen, and its learning was feasible for them."		
**22-ii) Highlight unanswered new questions, suggest future research**		
Other information		
**23) CONSORT: Registration number and name of trial registry**		
Clincialtrials.gov NCT05593978		
**24) CONSORT: Where the full trial protocol can be accessed, if available**		
"The data sets generated and analyzed during this study are available from the corresponding author on reasonable request."		
**25) CONSORT: Sources of funding and other support (such as supply of drugs), role of funders**		
There was no source of funding to report.		
**X26-i) Comment on ethics committee approval**		
**x26-ii) Outline informed consent procedures**		
**X26-iii) Safety and security procedures**		
**X27-i) State the relation of the study team towards the system being evaluated**		
